# Immunohematologic parallels and paradoxes: Africa vs Russia in the HIV era- a narrative review

**DOI:** 10.1097/MS9.0000000000004026

**Published:** 2025-10-07

**Authors:** Emmanuel Ifeanyi Obeagu

**Affiliations:** Department of Biomedical and Laboratory Science, Africa University, Zimbabwe

**Keywords:** ART, cytopenias, ethnoracial differences, HIV, immunohematology

## Abstract

Human Immunodeficiency Virus (HIV) infection significantly impacts the immune system, often resulting in various hematological complications, including anemia, neutropenia, and thrombocytopenia. These cytopenias are influenced by several factors, including the direct effects of HIV on the bone marrow and the side effects of antiretroviral therapy. However, there are notable ethnoracial differences in the immunohematological profiles of HIV-infected individuals, particularly between African and Russian populations. Africa, with its high prevalence of co-infections such as malaria, tuberculosis, and helminthiasis, presents unique challenges in managing HIV-related cytopenias. In contrast, in Russia, chronic comorbidities such as hepatitis and alcohol use significantly influence the immune and hematological responses to HIV. These differences underline the importance of considering both genetic and environmental factors when diagnosing and managing HIV-related hematological disorders. While both regions face substantial HIV burdens, the immunohematologic outcomes differ considerably due to regional co-morbidities and genetic predispositions. In Africa, malaria and helminth infections frequently exacerbate anemia, neutropenia, and thrombocytopenia, while in Russia, liver diseases, alcohol use, and chronic viral infections contribute to myelosuppression and hematological abnormalities. Moreover, certain genetic factors, such as Benign Ethnic Neutropenia in African populations, complicate the interpretation of hematological data. These disparities necessitate region-specific approaches to diagnosis, treatment, and management, tailored to address the unique challenges posed by each population’s distinct disease environment and health care system.

## Introduction

Human Immunodeficiency Virus (HIV) remains a global health challenge, with its impact on the immune system and hematological health being a critical concern^[1,2]^. The disease’s hallmark is the progressive depletion of CD4 + T-cells, leading to severe immunosuppression. One of the most significant complications of HIV infection is the development of various hematological abnormalities, including anemia, neutropenia, and thrombocytopenia. These conditions are often compounded by other health factors such as co-infections, co-morbidities, and genetic predispositions, which vary significantly across different populations^[3–5]^. This review explores the immunohematological profiles of HIV-infected individuals in two distinct regions – sub-Saharan Africa and Russia – highlighting the ethnoracial and environmental factors that influence the manifestation and management of HIV-related cytopenias. In sub-Saharan Africa, where the HIV epidemic is most pervasive, the burden of co-infections is notably high. Malaria, tuberculosis, and helminthic infections are common in the region and often present with overlapping symptoms with HIV, making diagnosis and treatment more complex^[6,7]^. These infections can exacerbate the hematological complications associated with HIV, especially anemia, neutropenia, and thrombocytopenia. For example, malaria-induced anemia is one of the most common hematological manifestations in HIV-infected individuals in Africa, with both malaria and HIV compromising red blood cell production and increasing the risk of severe anemia. The presence of these co-infections adds a layer of complexity to managing HIV, requiring clinicians to consider not only HIV treatment but also the management of other infectious diseases that directly impact the immune and hematological systems^[8–11]^.

**HIGHLIGHTS**

- Divergent Human Immunodeficiency Virus subtypes influence immune response dynamics across regions.
- Antiretroviral therapy access disparities shape immunologic recovery trends.
- Co-infections like tuberculosis impact hematologic profiles differently.
- Sociocultural factors affect disease progression.
- Paradoxical immune activation patterns highlight genetic and environmental interplay.

Russia, on the other hand, presents a different set of challenges for HIV-infected individuals. While the country has a lower prevalence of malaria and helminths compared to sub-Saharan Africa, it faces significant issues related to alcohol consumption, hepatitis C, and drug-resistant HIV strains. Chronic liver disease, particularly hepatitis C, has a substantial impact on platelet production, leading to thrombocytopenia in HIV patients. Additionally, alcohol abuse exacerbates myelosuppression, making anemia and neutropenia more common in Russian HIV-infected individuals. The interaction between HIV and these co-morbidities has led to unique hematological profiles, necessitating tailored therapeutic approaches to address the specific needs of this population^[12–16]^. Although both African and Russian populations face HIV-related cytopenias, the underlying mechanisms differ due to regional health challenges and genetic factors. African populations often exhibit a higher baseline of Benign Ethnic Neutropenia (BEN), a condition characterized by low neutrophil counts without clinical symptoms. This genetic predisposition complicates the diagnosis of neutropenia in HIV-infected African individuals, as lower neutrophil counts may not necessarily indicate an HIV-related complication. On the other hand, Russian populations often exhibit neutropenia and thrombocytopenia due to ART-induced bone marrow suppression and the additional burden of chronic diseases such as hepatitis and alcohol-related liver disease. These divergent immunohematologic responses underscore the importance of ethnoracial and environmental factors in shaping the clinical management of HIV^[17–19]^.

Immunohematological changes in HIV-infected individuals are not only a result of the direct effects of the virus on the immune and hematological systems but also a consequence of antiretroviral therapy (ART). ART has revolutionized the treatment of HIV, significantly improving survival rates and reducing the incidence of opportunistic infections. However, ART can have unintended effects on blood cell production, particularly in populations with pre-existing conditions like anemia or liver disease. In both Africa and Russia, ART regimens must be selected with care to minimize side effects, such as bone marrow suppression, that could worsen hematological complications. Moreover, the advent of drug-resistant HIV strains complicates treatment in both regions, necessitating ongoing research into optimizing ART regimens for diverse populations^[1,20,21]^. The understanding of immunohematological variations in African and Russian HIV-infected populations can also inform public health strategies. In Africa, where co-infections like tuberculosis and malaria are prevalent, health care systems need to integrate HIV treatment with the management of these diseases to improve overall health outcomes^[22,23]^. In Russia, where chronic viral hepatitis and alcohol abuse are more common, public health interventions should focus on reducing the burden of these conditions through harm reduction strategies and improved access to hepatitis treatment. Both regions require comprehensive, multi-disciplinary approaches to manage HIV-associated cytopenias effectively, with tailored treatment protocols that address local disease burdens and genetic factors.

## Aim

The aim of this review is to explore and analyze the immunohematological trends, challenges, and therapeutic implications for HIV-infected populations, with a particular focus on the contrasting contexts of Africa and Russia.

## Methods

This manuscript is a narrative review aimed at synthesizing current knowledge on immunohematologic manifestations of HIV infection in Africa and Russia. Literature searches were conducted using PubMed, Scopus, Web of Science, and Google Scholar databases for studies published up to 2025. Keywords included combinations of “HIV,” “hematology,” “anemia,” “leukopenia,” “thrombocytopenia,” “co-infections,” “Africa,” “Russia,” “ART,” and “immune activation.” Studies were selected based on thematic relevance, clinical significance, and quality of evidence. Both primary research studies (cohort, cross-sectional, and case-control) and high-quality narrative reviews were included to provide a comprehensive perspective. Articles focusing on non-HIV populations, unrelated hematologic conditions, or lacking regional specificity were excluded. Although this review does not follow systematic review protocols (e.g., PRISMA), care was taken to ensure broad coverage, balanced regional representation, and inclusion of clinically meaningful data. Data extraction focused on hematologic trends, co-infection profiles, ART-related complications, and immunologic outcomes. Findings were synthesized narratively, emphasizing region-specific patterns and comparative insights relevant to clinical practice and public health.

### Regional HIV epidemiology, ART access, and health care context

### Africa

Sub-Saharan Africa continues to bear the highest global HIV burden, accounting for approximately 67% of all people living with HIV worldwide. Prevalence varies by country, ranging from under 1% in some North African nations to over 20% in heavily affected Southern African regions. HIV-1 subtype C predominates in most of sub-Saharan Africa, influencing disease progression and ART response^[24,25].^ ART coverage has expanded significantly over the past decade due to large-scale public health initiatives and global funding programs. Despite this progress, challenges remain, including uneven access in rural areas, late HIV diagnosis, and interruptions in drug supply chains. Co-infections such as tuberculosis, malaria, and helminthiasis further complicate clinical management, often exacerbating hematologic abnormalities. Health care infrastructure varies widely across the continent, with limited diagnostic capacity and a high reliance on community-based care in resource-limited settings^[26]^.

### Russia

In contrast, Russia faces a concentrated but rapidly growing HIV epidemic, primarily affecting key populations such as people who inject drugs, men who have sex with men, and individuals with high-risk sexual behavior. National prevalence estimates suggest around 1% of the adult population is living with HIV, but certain urban and regional hotspots report rates exceeding 5%. HIV-1 subtypes A and B dominate, with subtype A particularly prevalent among injecting drug users, influencing treatment response and disease progression^[27,28]^. ART coverage in Russia has improved but remains limited by late diagnosis, stigma, inconsistent adherence, and regional disparities in drug availability. Harm reduction programs, including needle exchange and opioid substitution therapy, are variably implemented and often constrained by social and legal barriers. The health care system is characterized by centralized specialized care with limited integration of community-based services, which can delay early diagnosis and intervention. Co-morbidities such as hepatitis C virus (HCV) infection and alcohol-related liver disease further challenge hematologic and immunologic outcomes in these populations^[29]^.

### Comparative insights

This regional comparison highlights both parallels and divergences: while Africa faces a generalized epidemic with high prevalence and widespread co-infections, Russia contends with a concentrated epidemic driven by behavioral risk factors and comorbidities. ART access has expanded in both regions but remains limited by structural and social barriers, influencing immune recovery and hematologic outcomes. Understanding these epidemiologic, virologic, and health care context differences is critical for tailoring region-specific clinical strategies and public health interventions.

### HIV and immunohematology

The global burden of HIV remains one of the most pressing public health challenges of the 21st century, affecting millions of individuals worldwide. As the HIV pandemic continues to evolve, it has become increasingly evident that the virus’s impact extends far beyond the immune system, influencing a wide array of physiological processes, including hematology. HIV-induced immunosuppression, characterized by the depletion of CD4 + T-cells, renders individuals susceptible to a range of hematological abnormalities. These complications, which often manifest as anemia, neutropenia, and thrombocytopenia, complicate both the diagnosis and management of HIV, presenting a unique set of challenges for health care providers worldwide. However, the immunohematologic consequences of HIV are not uniform; they vary significantly across different populations due to factors such as co-infections, genetic predispositions, and environmental influences, all of which require nuanced, region-specific approaches to treatment^[24,25]^. In regions where HIV is most prevalent, such as sub-Saharan Africa, the intersection of HIV and hematology presents a particularly complex challenge. Co-infections, such as malaria, tuberculosis, and helminths, exacerbate the hematological complications associated with HIV, often making it difficult to distinguish between the effects of HIV and those of concurrent infections. Malaria, for example, can induce severe anemia and exacerbate the risk of opportunistic infections, particularly when individuals are already immunocompromised due to HIV. In addition, African populations may possess genetic traits that influence their hematological responses to HIV, such as Benign Ethnic Neutropenia (BEN), a condition in which individuals from certain ethnic backgrounds, particularly those of African descent, exhibit persistently low neutrophil counts without showing signs of infection or disease. These unique challenges require tailored treatment strategies that address not only HIV but also the multiple co-morbidities that are prevalent in these regions^[26]^.

On the other side of the globe, countries like Russia face a different set of immunohematologic hurdles related to HIV. While the prevalence of co-infections such as malaria is low, chronic diseases like hepatitis C, alcohol-related liver disease, and high rates of injecting drug use significantly affect the hematological health of HIV-infected individuals. Hepatitis C, for example, is a leading cause of liver dysfunction in Russia and can lead to thrombocytopenia due to impaired platelet production. Alcohol abuse, common among individuals living with HIV in Russia, further exacerbates liver damage, contributing to myelosuppression and increased hematological abnormalities. Additionally, the rising prevalence of drug-resistant HIV strains complicates the treatment landscape, making it crucial for health care providers to adopt personalized and more intensive ART regimens that account for these unique immunohematological challenges. As a result, the clinical management of HIV in Russia requires a careful balancing act to address the HIV infection alongside the impact of these chronic comorbidities^[27,28]^. The intersection of HIV and hematology is not confined to these regions but is a global phenomenon, and the challenges of managing immunohematologic complications are universally shared. Despite the differences in underlying causes and health burdens between regions like Africa and Russia, HIV-related cytopenias – such as anemia, neutropenia, and thrombocytopenia – are common across populations. These hematological manifestations are a direct consequence of the interplay between HIV’s effects on the immune system, the bone marrow, and the side effects of ART. While ART has been a game-changer in the fight against HIV, improving life expectancy and reducing morbidity, it is not without its drawbacks. ART-related bone marrow suppression can further exacerbate hematological disorders, especially in populations with pre-existing conditions. As ART regimens evolve to address the growing threat of drug-resistant HIV strains, it is crucial to consider the long-term effects of these therapies on hematological health^[29]^.

### Hematological manifestations in Africa: co-infection complications

In sub-Saharan Africa, HIV presents an extraordinary challenge not only because of its high prevalence but also due to the complex web of co-infections that often accompany it. The continent’s heavy burden of infectious diseases such as malaria, tuberculosis (TB), helminths, and HIV creates a unique health care dilemma. These co-infections do not merely coexist with HIV; they often exacerbate the disease’s impact on the immune system and hematological health. The confluence of these factors makes it essential for health care providers to consider the full spectrum of infections and their interactions when managing individuals living with HIV. The hematological complications that arise from this co-infection burden are particularly profound, with conditions like anemia, neutropenia, and thrombocytopenia becoming common and often intertwined with the effects of both HIV and its co-infections^[30,31]^.

Among the most significant hematological manifestations of co-infection in Africa is anemia. HIV alone can lead to anemia through several mechanisms, such as bone marrow suppression and the destruction of red blood cells. However, when malaria is added to the mix, the risk of anemia becomes even more pronounced. Malaria, caused by the Plasmodium parasites, is one of the leading causes of anemia in sub-Saharan Africa. The parasitic destruction of red blood cells and the inflammatory response triggered by the infection further complicate the already compromised hematological status of an individual living with HIV. The combination of malaria-induced anemia and HIV-induced immunosuppression often results in a clinical picture that is difficult to manage, requiring simultaneous treatment for both infections. This scenario is particularly challenging in settings where resources are limited, as the treatment regimens for HIV and malaria often need to be carefully coordinated to avoid drug interactions and complications^[32,33]^.

TB, another major co-infection in Africa, further complicates the hematological picture. TB is well known to cause anemia, particularly through chronic inflammation and the suppression of erythropoiesis (the production of red blood cells). In individuals with HIV, the situation becomes more complicated as the immune suppression caused by the virus increases the risk of developing active TB. The systemic inflammation induced by both HIV and TB can lead to the release of cytokines that suppress red blood cell production, exacerbating anemia. Moreover, the treatment for TB, particularly the use of anti-tuberculosis drugs like rifampicin, can also interact with ART, potentially leading to adverse effects on the bone marrow and further impairing hematological health. The dual challenge of managing HIV and TB – both of which impact the immune system and hematopoiesis – requires careful monitoring and an integrated approach to therapy that considers the pharmacological interactions and the heightened risk of adverse hematological outcomes^[34]^.

In addition to malaria and TB, helminthic infections are another significant contributor to the hematological complications in HIV-infected individuals in Africa. Helminths, such as hookworms and schistosomes, are prevalent in many African regions and can lead to chronic blood loss, further exacerbating anemia. The inflammatory responses triggered by these parasitic infections can also impair the function of the bone marrow, leading to neutropenia and thrombocytopenia in some cases. Moreover, these infections often go unnoticed due to their chronic and subtle nature, yet they compound the challenges of managing HIV, especially in populations that are already immunocompromised. The presence of helminthic infections in HIV-infected individuals can complicate the interpretation of hematological data, as the overlapping effects of parasitic infections and HIV-related immunosuppression require careful clinical evaluation to differentiate between the two^[35]^.

The clinical management of these overlapping infections is a delicate balancing act. Health care providers must not only treat HIV and its direct effects on the immune system but also address the co-infections that worsen hematological complications. The introduction of ART has dramatically improved the prognosis for HIV-infected individuals, helping to restore immune function and prevent the progression to AIDS. However, ART can also interact with the drugs used to treat co-infections, making the selection of appropriate medications a critical aspect of care. For instance, antiretroviral drugs like rifampicin may reduce the efficacy of certain ART regimens, particularly in the treatment of TB, requiring adjustments in medication dosages or the use of alternative therapies. Additionally, in areas where malaria is endemic, health care providers must carefully consider the potential for drug resistance and the impact of anti-malarial treatments on the immune system and hematological health^[36]^.

The burden of co-infections in HIV-infected individuals also has broader implications for public health in Africa. The widespread nature of these diseases, coupled with their overlapping clinical features, presents a major challenge for diagnostic accuracy and timely intervention. In many low-resource settings, access to diagnostic testing may be limited, making it difficult to distinguish between HIV-related cytopenias and those caused by co-infections. This can lead to delays in appropriate treatment and an increased risk of morbidity and mortality. Furthermore, the complexity of managing multiple infections simultaneously places significant strain on health care systems, particularly in regions with limited infrastructure and health care personnel. Efforts to improve diagnostic capacity, integrate care for HIV and co-infections, and ensure the availability of appropriate treatments are crucial steps in addressing the burden of these overlapping diseases^[37]^.

### Hematological manifestations in Russia: co-morbidities and alcohol use

In Russia, the landscape of HIV care is shaped by unique challenges that are not only related to the virus itself but also the interplay with co-morbidities, lifestyle factors, and public health issues prevalent in the region. One of the most significant factors influencing the hematological health of people living with HIV in Russia is the high prevalence of alcohol use, which significantly exacerbates the hematological complications associated with HIV. Alcohol consumption is widespread across many demographics in Russia, contributing to a range of health issues, including liver disease, cardiovascular conditions, and hematological abnormalities. This is compounded by other co-morbidities commonly seen in HIV-positive individuals, such as hepatitis C, which frequently co-exists with HIV and further complicates the management of hematological health^[38]^.

Alcohol use in Russia is a well-documented public health challenge, with high rates of consumption contributing to liver damage, including alcoholic liver disease (ALD) and cirrhosis. In individuals living with HIV, the liver is particularly vulnerable due to the combined effect of alcohol-induced toxicity and HIV’s impact on immune function. Chronic liver disease, particularly when exacerbated by alcohol, leads to thrombocytopenia (low platelet count), which is one of the most common hematological manifestations in this population. The liver’s impaired ability to produce clotting factors also contributes to increased bleeding risk. Furthermore, alcohol-induced liver dysfunction can worsen the immunosuppressive effects of HIV, making individuals more susceptible to opportunistic infections that can further compromise their hematological health. These intertwined effects make the clinical management of HIV-infected individuals in Russia particularly complex, requiring a holistic approach that addresses both the HIV infection and the consequences of alcohol abuse^[20]^.

Co-morbidities such as hepatitis C also play a crucial role in the hematological manifestations of HIV in Russia. Hepatitis C is a common co-infection among individuals with HIV in the region, particularly among people who inject drugs, which further complicates the clinical management of the disease. Hepatitis C leads to liver damage, including fibrosis and cirrhosis, and can cause thrombocytopenia and anemia. These hematological disorders are often compounded by the bone marrow suppression caused by HIV itself. The interaction between hepatitis C, alcohol use, and HIV creates a perfect storm for the development of severe hematological abnormalities. In many cases, the combination of these factors can lead to the need for complex therapies, including ART to manage HIV, antivirals to treat hepatitis C, and interventions to manage alcohol-related liver disease. This multi-layered treatment approach poses significant challenges, particularly in terms of drug-drug interactions and the increased risk of adverse effects on the liver and bone marrow^[39]^.

The hematological consequences of HIV, alcohol use, and co-morbidities in Russia also extend to the management of anemia. Anemia is a common condition among individuals with HIV, arising from multiple factors, including chronic inflammation, poor nutrition, and bone marrow suppression caused by the virus itself. However, in the context of alcohol use and hepatitis C, anemia can be particularly severe. Alcohol-induced damage to the bone marrow, along with the chronic viral infection from hepatitis C, further diminishes red blood cell production. The use of ART to manage HIV, while life-saving, can also contribute to hematological abnormalities, including anemia. Certain antiretroviral drugs, such as zidovudine, are known to cause bone marrow suppression, which may further exacerbate the anemia in individuals already affected by co-morbidities. In this complex clinical picture, the challenge lies not only in managing HIV but also in addressing the underlying alcohol use and co-morbidities that contribute to these hematological manifestations^[38]^.

The management of hematological complications in HIV-infected individuals with alcohol use and co-morbidities in Russia requires a careful, multi-disciplinary approach. Treatment must involve not only the use of ART but also interventions to address alcohol use disorder and co-infections such as hepatitis C. This often requires a combination of medications, counseling, and lifestyle modifications. The first line of therapy should aim to reduce alcohol consumption through behavioral interventions and pharmacotherapy, as this can significantly reduce the burden of alcohol-related liver damage and improve overall health outcomes. For hepatitis C co-infection, the introduction of direct-acting antivirals (DAAs) has revolutionized the treatment landscape, providing an opportunity to cure the infection and reduce the risk of further liver damage. ART regimens should also be selected with caution to minimize bone marrow suppression and other hematological side effects. A holistic approach to managing HIV in the context of alcohol use and co-morbidities not only improves hematological health but also enhances the overall well-being and quality of life for individuals living with HIV in Russia^[20]^.

In addition to the clinical challenges, there are significant public health concerns related to the high rates of alcohol use in Russia. Alcohol abuse in the general population contributes to a range of chronic health conditions, including liver disease, cardiovascular disease, and mental health disorders. The intersection of alcohol use and HIV presents a unique challenge for health care providers, particularly in resource-limited settings where access to specialized care may be limited. The stigma associated with both HIV and alcohol abuse may also deter individuals from seeking care, further exacerbating the public health burden. Public health interventions aimed at reducing alcohol consumption, improving access to HIV care, and addressing co-morbidities such as hepatitis C are critical in improving the health outcomes for individuals living with HIV in Russia. Increased awareness and education about the impact of alcohol use on HIV progression and hematological health could lead to better early diagnosis and treatment, ultimately improving the quality of care for this vulnerable population^[39]^.

### Comparative immunohematological trends

The intersection of immunohematology and HIV presents distinct challenges and trends across different regions, shaped by genetic, environmental, and health care system factors. Africa and Russia, two regions burdened by the HIV epidemic, display significant differences in the way immunohematological issues manifest in HIV-infected individuals. These variations in hematological outcomes – such as anemia, thrombocytopenia, and neutropenia – are influenced by factors unique to each region, including the prevalence of co-infections, lifestyle factors, genetics, and the availability of medical care. In Africa, the immunohematological impact of HIV is often compounded by the high prevalence of co-infections such as malaria, TB, and helminths. These infectious diseases, which are endemic to the region, exacerbate the hematological complications of HIV, leading to conditions like anemia, neutropenia, and thrombocytopenia. Malaria, in particular, is a leading cause of anemia in sub-Saharan Africa, and its interaction with HIV-induced immunosuppression can result in more severe outcomes. The cytokine responses from malaria infection also have a significant impact on the bone marrow, which is already compromised by HIV, further impairing hematopoiesis. Additionally, helminthic infections, which are widespread in Africa, contribute to chronic blood loss and inflammation, compounding the anemia seen in HIV-infected individuals. These infections not only increase the burden of hematological disorders but also affect the immune system, making it harder to manage HIV effectively. Treatment regimens in Africa must thus address the multifactorial nature of these immunohematological issues, with integrated care that targets both HIV and its co-infections^[40–42]^.

In contrast, Russia’s immunohematological landscape in the HIV era is shaped by a different set of challenges. Although co-infections such as tuberculosis and hepatitis C are common, the primary exacerbating factor in Russia is the high rate of alcohol use, which contributes to a range of hematological complications in HIV-infected individuals. Alcohol abuse leads to liver damage, including cirrhosis and ALD, which further impairs hematopoiesis, leading to thrombocytopenia and anemia. The immunosuppressive effects of alcohol, when combined with HIV-related immune dysfunction, create a dual challenge in managing hematological health. Hepatitis C, often co-infected with HIV, also significantly impacts the immune system and the liver, compounding hematological abnormalities like thrombocytopenia. Furthermore, the bone marrow suppression caused by both HIV and alcohol abuse requires careful monitoring and adjustment of ART to minimize side effects like anemia and neutropenia. In Russia, the focus on managing alcohol use disorder alongside HIV treatment is a critical element of care, with an emphasis on behavioral interventions and medications that address both alcohol dependence and viral infections^[43,44]^.

One of the key differences between Africa and Russia lies in the health care systems and access to treatment. In Africa, the widespread availability of ART has dramatically improved outcomes for HIV-infected individuals, but the simultaneous burden of infectious diseases presents a unique challenge. In contrast, while ART is widely available in Russia, the health care system faces challenges related to alcohol use disorder and the co-management of chronic diseases like hepatitis C. Both regions face the difficulty of managing the interactions between ART and other medications used to treat co-infections or alcohol-induced liver disease. For example, drugs like rifampicin used to treat tuberculosis can reduce the efficacy of certain ART regimens, while the interaction between ART and alcohol or hepatitis C treatments requires careful consideration to avoid exacerbating hematological complications. In both contexts, multidisciplinary care teams that include infectious disease specialists, hematologists, and addiction counselors are essential to optimize treatment outcomes and manage the complex interplay between HIV, alcohol, and co-infections^[33,45,46]^.

From an immunological standpoint, Africa and Russia show stark differences in the genetic susceptibility to HIV-related hematological complications. In Africa, certain genetic factors, including variations in blood group antigens and hemoglobinopathies like sickle cell disease and thalassemia, may influence how individuals respond to both HIV infection and its hematological manifestations. These genetic factors can impact red blood cell survival and the immune response, potentially leading to altered rates of anemia and other hematological issues. Conversely, Russia’s immunohematological trends are influenced by a genetic background that may be more prone to chronic liver diseases, especially those exacerbated by alcohol use. The higher prevalence of genetic mutations related to liver metabolism and alcohol processing could explain the more prominent liver-related hematological complications in Russia. These genetic differences underline the need for tailored diagnostic and therapeutic strategies that consider the genetic profile of HIV-infected individuals in both regions^[47,48]^. Another important aspect of the comparative analysis between Africa and Russia is the influence of lifestyle and socioeconomic factors. In Africa, malnutrition and poor access to health care often exacerbate the hematological consequences of HIV^[49]^.

The combination of inadequate nutrition, frequent infections, and a high burden of HIV-related stigma all contribute to the progression of hematological disorders. Conversely, in Russia, the socio-economic burden of alcohol-related diseases, coupled with a high smoking rate, contributes to a different set of challenges in HIV management^[50]^. The widespread use of alcohol and tobacco affects the cardiovascular and hematological systems, creating a high risk for conditions like thrombocytopenia, anemia, and cardiovascular complications. These lifestyle-related factors necessitate a more comprehensive approach to care in Russia, with an emphasis on reducing alcohol consumption, improving diet, and addressing smoking as part of a holistic HIV care regimen (Table 1)^[51]^.

**Table 1:** Comparing Hematological Trends, Key Co-infections, and ART-related complications in HIV populations from Africa and Russia

Parameter	Africa	Russia	Notes/Comments
Anemia prevalence	40–70%	25–50%	Higher prevalence in Africa due to nutritional deficiencies, malaria, TB, and chronic inflammation
Leukopenia/Neutropenia	Common; often accompanied by eosinophilia and monocytosis	Moderate; neutropenia mainly linked to ART toxicity and co-infections	African populations show parasite-driven immune modulation; Russian populations show bacterial/HCV-driven effects
Thrombocytopenia	10–30%; often transient	10–25%; more chronic and ART-related	Immune-mediated in both regions; African cases often linked to acute infections
Key co-infections	Malaria, Tuberculosis (TB), Helminths	Hepatitis C (HCV), Hepatitis B (HBV), Alcohol-related liver disease (ALD), bacterial infections	Co-infections modulate immune activation and hematologic outcomes
ART coverage	Increasing; substantial scale-up, though access varies by country	Moderate; late diagnosis and adherence issues common	ART improves hematologic parameters but zidovudine-containing regimens may induce cytopenias
ART-related complications	Myelosuppression (mainly zidovudine), macrocytosis	Myelosuppression, anemia, thrombocytopenia, hepatotoxicity	Side effects more evident in Russian cohorts due to late ART initiation and polypharmacy
Immune activation markers	Elevated IL-6, CRP, sCD14; paradoxically higher CD4 recovery despite higher viremia	Elevated IL-6, CRP, sCD14; slower CD4 recovery	Reflects differences in early-life microbial exposure, co-infections, and immune resilience
Genetic/host factors	Benign Ethnic Neutropenia common; other polymorphisms may confer hematologic resilience	Less prevalent; host genetics contribute less to resilience	Explains some regional differences in cytopenia severity and recovery

### Ethnoracial and environmental influences on immunohematology

Immunohematology, the study of blood disorders from an immunological perspective, is significantly shaped by both ethnoracial and environmental factors. These influences play a vital role in the prevalence, progression, and management of hematological disorders, particularly in the context of global diseases such as HIV. The intricate relationship between genetics, environment, and immunological responses varies across populations and geographic regions, contributing to distinct immunohematological trends. Ethnoracial differences in immunohematology have been well documented, as genetic factors often influence the immune system’s ability to respond to various infections, as well as the manifestation and severity of hematological diseases^[52,53]^. For instance, individuals of African descent have a higher prevalence of sickle cell disease, a genetic disorder that affects red blood cells, causing them to become rigid and sickle-shaped under low oxygen conditions. This genetic mutation provides some protection against malaria, a disease endemic to many regions of sub-Saharan Africa, but it also predisposes these individuals to severe anemia, thrombocytopenia, and other hematological complications. In contrast, populations in Russia are less likely to have such genetic predispositions, though they may exhibit higher rates of liver disease and alcohol-induced hematological disorders, partly due to different genetic backgrounds related to alcohol metabolism. These ethnoracial distinctions highlight the importance of considering genetic diversity when diagnosing and treating immunohematological conditions, especially in regions with significant population heterogeneity^[54,55]^.

Environmental factors also exert a profound influence on immunohematology, as the physical surroundings in which individuals live can significantly impact both immune and hematological health. In regions where, infectious diseases such as malaria, tuberculosis, and helminths are prevalent, these environmental factors can exacerbate the effects of HIV and other blood-related disorders. For example, the chronic inflammation and blood loss associated with malaria infections can compound the anemia seen in HIV-positive individuals, especially in sub-Saharan Africa. Similarly, the burden of other parasitic diseases may lead to a sustained immune response that further impairs the production of blood cells. In contrast, in Russia, where co-infections like hepatitis C and alcohol use disorder are more prevalent, the environment contributes to distinct immunohematological challenges. Alcohol abuse, a widespread social issue, induces liver damage that affects platelet production, exacerbates anemia, and compromises immune function. Moreover, the harsh climatic conditions in some areas of Russia, combined with high rates of smoking and poor nutrition, can lead to increased susceptibility to hematological disorders such as thrombocytopenia and neutropenia^[56,57]^.

Together, these ethnoracial and environmental influences create a complex landscape for managing immunohematological conditions. In populations with a high genetic susceptibility to certain hematological disorders, such as those with sickle cell anemia, public health efforts are often focused on improving early diagnosis and providing appropriate treatments to manage crises and prevent complications. In areas where environmental factors exacerbate immunohematological disorders, such as malaria or alcohol abuse, health care systems may focus on integrated care models that address both infectious diseases and lifestyle factors, alongside HIV treatment. The interplay between genetic susceptibility, environmental exposures, and public health infrastructure underscores the need for a comprehensive, region-specific approach to managing immunohematological disorders. Public health policies, access to quality care, and health education must all be adapted to account for the unique needs of diverse populations, ensuring that interventions are both effective and culturally appropriate^[58,59]^.

### Diagnostic and therapeutic implications in immunohematology

The diagnostic and therapeutic approaches to immunohematological disorders are deeply influenced by both the genetic and environmental contexts of the populations being treated. As health care systems across the world strive to manage a diverse array of blood disorders, understanding the diagnostic challenges and therapeutic implications becomes crucial. This is particularly important in the context of HIV, where hematological complications such as anemia, thrombocytopenia, and neutropenia are common. These complications can be exacerbated by genetic predispositions, co-infections, and environmental factors, leading to a complex interplay of influences that health care professionals must navigate. Tailoring diagnostic and therapeutic strategies to the unique needs of individuals and populations is essential for improving outcomes and optimizing treatment^[59,60]^. In regions such as sub-Saharan Africa, where malaria and other parasitic diseases are endemic, the overlap between HIV-related anemia and anemia caused by these infections can present significant challenges. Diagnosing anemia, for instance, requires distinguishing between the direct effects of HIV, such as bone marrow suppression and immune dysfunction, and the secondary effects of co-infections like malaria, helminths, or tuberculosis. This can involve the use of advanced diagnostic tools, including PCR testing to detect specific pathogens, as well as comprehensive blood panels to assess red blood cell counts, hemoglobin levels, and platelet function. In contrast, in areas such as Russia, where alcohol use disorder and co-infection with hepatitis C are more common, diagnostic strategies must also include screening for liver function abnormalities and monitoring platelet counts and liver enzyme levels. In both cases, accurate diagnosis is fundamental to distinguishing between primary HIV-related hematological issues and secondary complications arising from environmental factors, co-infections, or lifestyle choices^[61,62]^.

Therapeutically, the treatment of immunohematological disorders in HIV-positive individuals requires careful consideration of the underlying cause, the patient’s overall health, and the regional context. In Africa, where co-infections like malaria and tuberculosis are prevalent, effective treatment often necessitates a multi-disciplinary approach. For example, addressing anemia in an HIV-positive individual in a malaria-endemic region might involve antimalarial treatment alongside ART, while also managing any underlying nutritional deficiencies or parasitic infections. The therapeutic approach must also consider the potential drug interactions between ART and medications used to treat co-infections, such as rifampicin for tuberculosis, which can reduce the effectiveness of certain antiretrovirals. In regions like Russia, where alcohol-induced liver disease is a common co-morbidity in HIV patients, the management of hematological complications may require a combination of ART, alcohol cessation programs, and hepatoprotective therapies. In these cases, managing liver function and platelet levels becomes paramount to prevent conditions like thrombocytopenia and bleeding disorders. In both settings, ongoing monitoring is essential to adjust treatments as needed and ensure optimal therapeutic outcomes^[63,64]^.

Furthermore, the therapeutic implications of immunohematological disorders extend to the use of blood transfusions and other supportive treatments. In regions where anemia is a frequent complication, especially in individuals with HIV and malaria, blood transfusions may be necessary to restore normal hemoglobin levels and prevent severe complications such as organ failure. However, the use of blood transfusions comes with its own set of challenges, particularly in resource-limited settings. Issues such as blood availability, the risk of transmission of other infections, and the need for careful monitoring of transfusion reactions must all be addressed. In contrast, in more developed regions like Russia, where alcohol abuse may contribute to anemia and thrombocytopenia, the therapeutic focus may shift more toward managing the root cause of the hematological disorder, such as addressing liver damage and providing treatments to manage alcohol dependence. Here, the use of blood transfusions may be less common, and the focus may be on preventing further damage to the bone marrow and improving the patient’s overall liver health^[65,66]^.

Ultimately, the diagnostic and therapeutic implications of immunohematological disorders in HIV-infected individuals highlight the need for region-specific strategies that consider both the biological and environmental factors that influence health outcomes. In Africa, addressing the complex interplay between HIV and co-infections requires a multi-faceted approach that includes not only ART but also targeted treatments for infectious diseases and nutritional support^[67,68]^. In contrast, in Russia, managing alcohol use disorder and its hematological consequences necessitates a more focused approach that includes liver protection, behavioral therapy, and careful ART management^[69,70]^. Across both regions, however, the importance of integrated care cannot be overstated. A holistic approach that combines advanced diagnostics, effective therapeutic strategies, and patient education is essential to managing immunohematological complications and ensuring the best possible outcomes for HIV-infected individuals.

## Conclusion

HIV infection exerts profound immunohematologic effects, including anemia, leukopenia, and thrombocytopenia, across diverse populations. Africa and Russia, despite contrasting epidemiologic and health care landscapes, share overlapping hematologic patterns while displaying paradoxical differences in immune resilience, co-infection profiles, and ART responses. African cohorts often demonstrate higher baseline CD4 counts and hematologic recovery despite high viral loads, likely influenced by endemic infections, genetic factors, and early-life microbial exposure. In contrast, Russian populations experience more chronic cytopenias and slower immune recovery, compounded by late diagnosis, ART adherence challenges, and prevalent co-morbidities such as HCV and alcohol-related liver disease. These findings underscore the importance of context-specific monitoring, personalized ART strategies, and regionally tailored public health interventions. Future research should focus on integrating host genetics, co-infection patterns, and socioeconomic determinants to optimize hematologic outcomes and reduce HIV-related morbidity. By elucidating these immunohematologic parallels and paradoxes, this review provides a foundation for region-informed clinical management and global HIV policy planning.

## Data Availability

Not applicable as this a perspective.

## Research Registration Unique Identifying Number (UIN)

Not applicable as this a perspective.
